# Perineal injuries and birth positions among 2992 women with a low risk pregnancy who opted for a homebirth

**DOI:** 10.1186/s12884-016-0990-0

**Published:** 2016-07-29

**Authors:** Malin Edqvist, Ellen Blix, Hanne K. Hegaard, Olöf Ásta Ólafsdottir, Ingegerd Hildingsson, Karen Ingversen, Margareta Mollberg, Helena Lindgren

**Affiliations:** 1 Institute of Health and Care Sciences, The Sahlgrenska Academy, University of Gothenburg, Arvid Wallgrens backe hus 1, Box (PO) 457, 405 30 Gothenburg, Sweden; 2 Research Group: Maternal, Reproductive and Children’s Health, Faculty of Health Sciences, Oslo and Akershus University College of Applied Sciences, Oslo, Norway; 3 Research Unit, Women’s and Children’s Health, Juliane Marie Center for Women, Children and Reproduction, Copenhagen University Hospital, Rigshospitalet, Copenhagen, Denmark; 4 Department of Midwifery, Faculty of Nursing, University of Iceland, Reykjavík, Iceland; 5 Department of Nursing, Mid Sweden University, Sundsvall, Sweden; 6 Department of Women’s and Children’s Health, Uppsala University, Uppsala, Sweden; 7 Homebirth Association Sealand, Copenhagen, Denmark; 8 Department of Women’s and Children’s Health, Karolinska Institute, Stockholm, Sweden

**Keywords:** Home birth, Birth positions, Severe perineal trauma, Perineal injuries, Episiotomy, Waterbirth

## Abstract

**Background:**

Whether certain birth positions are associated with perineal injuries and severe perineal trauma (SPT) is still unclear. The objective of this study was to describe the prevalence of perineal injuries of different severity in a low-risk population of women who planned to give birth at home and to compare the prevalence of perineal injuries, SPT and episiotomy in different birth positions in four Nordic countries.

**Methods:**

A population-based prospective cohort study of planned home births in four Nordic countries. To assess medical outcomes a questionnaire completed after birth by the attending midwife was used. Descriptive statistics, bivariate analysis and logistic regression were used to analyze the data.

**Results:**

Two thousand nine hundred ninety-two women with planned home births, who birthed spontaneously at home or after transfer to hospital, between 2008 and 2013 were included. The prevalence of SPT was 0.7 % and the prevalence of episiotomy was 1.0 %. There were differences between the countries regarding all maternal characteristics. No association between flexible sacrum positions and sutured perineal injuries was found (OR 1.02; 95 % CI 0.86–1.21) or SPT (OR 0.68; CI 95 % 0.26–1.79). Flexible sacrum positions were associated with fewer episiotomies (OR 0.20; CI 95 % 0.10–0.54).

**Conclusion:**

A low prevalence of SPT and episiotomy was found among women opting for a home birth in four Nordic countries. Women used a variety of birth positions and a majority gave birth in flexible sacrum positions. No associations were found between flexible sacrum positions and SPT. Flexible sacrum positions were associated with fewer episiotomies.

## Background

Perineal injuries and severe perineal trauma involving the anal sphincter complex (SPT) are associated with short- and long-term morbidity, such as perineal pain [1, 2], dyspareunia [2, 3] and anal incontinence [4]. Both short- and long-term symptoms have an impact on women’s daily lives [5] and on women’s quality of life for those with persistent defects [6]. The prevalence of perineal injuries of all types is reported to be 77–86 % [7, 8] of which 60 % need to be sutured [8]. The incidence of SPT in the Nordic countries (in this article ‘Nordic countries’ refers to Norway, Sweden, Denmark and Iceland) varies from 2.3 % in Norway to 4.2 % in Denmark [9–11] whereas there is no national data available regarding the prevalence of less severe injuries.

Known risk factors for perineal trauma, including SPT are primiparity [12, 13], high birth weight [12] and occiput posterior presentation [14]. Obstetrical factors associated with SPT are a prolonged second stage [12, 15], instrumental delivery [16], episiotomy [17], poor visualization of the perineum [16], fundal pressure [12], the lithotomy position [18] and oxytocin augmentation [19]. Few studies have assessed risk factors for less severe perineal trauma such as second degree tears but the risk factors appears to be similar [20]. Home births have been associated with fewer perineal injuries and SPT compared to hospital births [21–23].

Women who choose home birth are a selected and highly motivated population. Generally they are multiparous, are older, and tend to have a higher socioeconomic status [24]. Fewer are smokers and overweight, which can be viewed as indicators of health [25]. The prevalence of planned home birth varies in the Nordic countries. In Sweden and Norway it is 0.06 % and 0.019 % respectively, while home birth is more common in Denmark and Iceland with 1.5–1.8 % [26]. It is not known whether the observed benefit of opting for a home birth with regard to SPT and perineal injuries is due to differences in midwifery practice, the selected population of women or other factors, such as birth position. Midwifery care measures at home to prevent perineal injuries include getting to know the woman before the onset of labor, following the physiological process of birth and letting her choose the position for birth [27]. Furthermore some of the obstetrical risk factors of SPT are not present in the home birth setting, such as instrumental delivery, the lithotomy position for birth, and oxytocin augmentation since the woman will be transferred to hospital in the event of an emergency or slow progress of labor. Waterbirth on the other hand is common in this setting [28]; in some studies it is associated with SPT and perineal injuries [23, 29] but not in others [30].

There is still controversy around whether upright or recumbent birth positions are beneficial or harmful with regard to SPT as well as less severe perineal injuries. Giving birth in the lateral and all-fours position has been associated with a higher prevalence of intact perineum [31–33] but this is not found in the meta-analysis by Gupta et al. [34]. Upright birth positions occur more often within certain birth settings, such as birth centers and at home [28]. Upright birth positions in Western obstetrics may be defined as positions in which a line connecting the center of a woman’s third and fifth vertebrae is more vertical than horizontal [34, 35]. According to this definition sitting, squatting, the birth-seat, kneeling and standing are defined as upright positions, whereas lateral and all-fours, semi-recumbent and the lithotomy position are considered supine positions [34], although they are different and may facilitate or hinder physiological birth.

Another possible definition is to classify birth positions in which the body weight is on or off the sacrum. Positions that take the weight off the sacrum and allow the pelvic outlet to expand might be favorable to facilitating spontaneous birth [36]. Birth positions that take the weight off the sacrum and could be categorized as flexible sacrum positions are kneeling, standing, all-fours, lateral position, squatting and giving birth on the birth seat. On the other hand all the positions where the woman is sitting or lying on her back, such as the supine and the semi-recumbent position put weight on the sacrum and could be categorized as non-flexible sacrum positions. The evidence as to the impact of upright birth and flexible sacrum positions on perineal outcomes remains inconclusive [36] and has to our knowledge not been tested in the home birth setting. Since home births are seldom recorded in the registers in the Nordic countries, the prevalence of perineal injuries, SPT, episiotomy and birth positions for women opting for a home birth is not known.

The objective of this study is to describe the prevalence of perineal injuries of different severity in a low-risk population of women who planned to give birth at home in four Nordic countries and to compare the prevalence of perineal injuries, SPT and episiotomy in flexible and non-flexible birth positions.

## Method

### Design and study sample

This is a prospective cohort study collecting data from planned home births in Norway, Denmark, Sweden and Iceland between 2008 and 2013. All midwives attending home births were asked to recruit their clients to the study. The women were given information about the study during pregnancy, and signed a form agreeing to participate. The method and data collection has been described previously by Blix et al. [37]

### Data collection

The data collection lasted from January 1^st^ 2008 to December 31^st^ 2012 in Norway, in Sweden from January 1^st^ 2009 to December 31^st^ 2013, in Denmark from March 1^st^ 2010 to May 15^th^ 2013 and in Iceland from January 1^st^ 2010 to December 31^st^ 2013.

The questionnaire included information about women’s background characteristics (country of residence, age, parity, marital status, Body Mass Index, tobacco use) (Table 1) and was completed by the attending midwife 1 week after the birth. The questionnaire also contained information about place of birth (home, during transfer, hospital) and birth outcome related to the woman [37]. All births that were planned to take place at home and started at home are included in the cohort irrespective of where the baby actually was born, at home or after transfer to the hospital. Birth positions were assessed according to type of position at the moment when the baby was born. Eight different positions were predefined. The positions that aim at expanding the pelvic outlet and taking weight off the sacrum are defined as flexible sacrum positions in this study. Birth positions were dichotomized into two groups: flexible or non-flexible sacrum positions. Positions that take the weight off the sacrum are: kneeling, standing, all-fours, squatting, the birth seat and lateral. Positions defined as non-flexible are semi-recumbent, lithotomy and supine positions. Perineal injuries were reported as sutured injury or not, episiotomy and SPT. A non-sutured injury includes no tear at all, small abrasions or minor injuries, which the midwife considered did not require suturing. A variable was created to capture total recorded perineal injuries, where SPT, episiotomy and sutured injuries were included.

**Table 1:** Socio-demographic background

	Total	Norway	Sweden	Denmark	Iceland	Chi^2^test
	*N* = 2992	*N* = 468	*N* = 438	*N* = 1799	*N* = 287	*p*-value
	n(%)	n (%)	n (%)	n (%)	n (%)	
Age groups						<0.001
<25 years	202 (6.8)	26 (5.6)	24 (5.5)	119 (6.6)	33 (11.5)	
25-34 years	1923 (64.3)	295 (63.0)	234 (53.4)	1188 (66.0)	206 (72.0)	
>35 years	850 (28.4)	145 (31.0)	177 (40.4)	481 (26.7)	47 (16.4)	
Missing	17 (0.6)	2 (0.4)	3 (0.7)	11 (0.6)	1 (0.3)	
Marital status						<0.001
Married/cohabit	2918 (97.5)	449 (95.7)	407 (92.9)	1779 (98.9)	284 (99.0)	
Not married/cohabit	51 (1.7)	17 (3.6)	13 (3.0)	20 (1.1)	1 (0.3)	
Missing	23 (0.8)	3 (0.6)	18 (4.1)	0	2 (0.7)	
Tobacco use						<0.001
Yes	198 (6.6)	16 (3.4)	5 (1.1)	167 (9.3)	10 (3.5)	
No	2735 (91.4)	450 (96.2)	425 (97.0)	1587 (88.2)	273 (95.1)	
Missing	59 (2.0)	2 (0.4)	8 (1.8)	45 (2.5)	4 (1.4)	
Number of children						0.004
First baby	524 (17.5)	80 (17.1)	70 (16.0)	313 (17.4)	61 (21.3)	
One previous child	1257 (42.0)	175 (37.4)	208 (47.5)	753 (41.9)	121 (42.2)	
Two previous children	828 (27.7)	137 (29.3)	113 (25.8)	494 (27.5)	84 (29.3)	
Three or more previous children	322 (10.8)	74 (15.8)	46 (10.5)	182 (10.1)	20 (7.0)	
Missing	61 (2.0)	2 (0.4)	1 (0.2)	57 (3.2)	1 (0.3)	
Body Mass Index (BMI), mean (SD)						
BMI-groups						0.001
<18.5	101 (3.4)	16 (3.4)	16 (4.7)	60 (3.3)	9 (3.1)	
18,5-24.9	1943 (64.9)	289 (61.8)	260 (59.4)	1220 (67.8)	174 (60.6)	
25.0-29.9	516 (17.2)	87 (18.6)	51 (11.6)	323 (18.0)	55 (19.9)	
>30	196 (6.6)	25 (5.3)	17 (3.9)	116 (6.4)	38 (13.2)	
Missing	236 (7.9)	51 (10.9)	94 (21.5)	80 (4.4)	11 (3.8)	

### Analysis

Descriptive statistics, Chi^2^ and ANOVA tests were used to present the background characteristics and compare data between the Nordic countries. The outcome variables were sutured perineal and vaginal injuries, SPT, episiotomies and total posterior trauma. Crude and adjusted odds ratios with a 95 % confidence interval were calculated between the outcome variables and flexible sacrum positions. Potential confounders were adjusted for using logistic regression. The IBM SPSS software package version 22.0 was employed for the data analysis.

## Results

For the purpose of this study, a selected sample of 2992 of the original cohort of 3068 women with a planned home birth was included. A total of 76 women who had a caesarean section or an instrumental delivery after transfer to hospital were excluded. Instrumental deliveries were excluded since they are performed in a supine or recumbent birth position. Of the 2992 women, 2796 (93.4 %) successfully gave birth at home and 196 (6.6 %) gave birth spontaneously after transfer to the hospital. The most common reason for transfer was slow progress of labor.

Table 1 shows the background characteristics of the 2992 women included in this study. The majority of the planned home births in this study occurred in Denmark (*n* = 1799), followed by Norway (*n* = 468), Sweden (*n* = 438) and Iceland (*n* = 287). The mean age for the total sample was 32 years (range 18–47). There were significant differences between the countries regarding all maternal characteristics. The Icelandic mothers were the youngest and the Swedish mothers were the oldest. The highest proportion of planned home births for women expecting their first baby occurred in Iceland (21.7 %) and in Denmark (18.5 %). Most women irrespective of country were married or cohabiting, did not smoke and were expecting their second baby. Among the multiparous women in the cohort, 140 (4.7 %) women attempted a planned home birth after a caesarean section (VBAC). The vast majority (85 %) of these planned VBAC home births occurred in Denmark.

The prevalence of SPT was 0.7 % for the total study population, 2.3 % among primiparas and 0.3 % among multiparas (Table 2). The only risk factors for SPT found in this study were primiparity (adj OR 9.90; CI 95 % 3.63–26.98) and birth weight > 4000 g (adj OR 2.87; CI 95 % 1.07–7.75) (Table 4). The overall prevalence of sutured injuries was 41.5 % (Table 2). When stratifying for parity, 60.9 % of the primiparous women had injuries considered as needing sutures and so did 36.8 % of the multiparous women. The prevalence of episiotomy was 1.0 %. The women who were transferred to hospital were more likely to have an episiotomy (OR 3.98; CI 95 % 1.72–9.22) (Table 4).

**Table 2:** Birth outcomes

	Total	Primiparas	Multiparas^a^	Missing
	*N* = 2992	*N* = 524	*N* = 2422	*N* = 46 (1.5)
	n (%)	n (%)	n (%)	
Birth weight				
<2999 g	134 (4.5)	44 (8.4)	88 (3.6)	
3000-3999 g	2029 (67.8)	392 (74.8)	1609 (66.4)	
4000-4499 g	607 (20.3)	55 (10.5)	541 (22.3)	
>4500 g	143 (4.8)	15 (2.9)	127 (5.2)	
Missing	79 (2.6)	18 (3.4)	57 (2.4)	
Sutured injury				
Yes	1242 (41.5)	319 (60.9)	891 (36.8)	
No	1709 (57.1)	196 (37.4)	1499 (61.9)	
Missing	41 (1.4)	9 (1.7)	32 (1.3)	
OASIS				
Yes	21 (0.7)	12 (2.3)	7 (0.3)	
No	2937 (98.2)	501 (95.6)	2392 (98.8)	
Missing	34 (1.1)	11 (2.1)	23 (0.9)	
Episiotomy				
Yes	31 (1.0)	23 (4.4)	7 (0.3)	
No	2926 (97.8)	493 (94.1)	2388 (98.6)	
Missing	35 (1.2)	8 (1.5)	27 (1.1)	
Total perineal injury				
Yes	1276 (42.6)	342 (65.3)	899 (37.1)	
No	1669 (55.8)	174 (33.2)	1484 (61.3)	
Missing	47 (1.6)	8 (1.5)	39 (1.6)	

^a^141 women with one previous CS, 3 women with 2 previous CS

Women gave birth in a variety of positions (Table 3). The majority (65.2 %) used flexible sacrum positions. Kneeling was the most frequently used birth position of the flexible sacrum positions regardless of parity (24.6 %). However for primiparous women semi-recumbent, which is considered as a non-flexible sacrum position was the most common position for birth (29.6 %), followed by kneeling (19.1 %) (Table 3). The prevalence of waterbirth was 31.8 % (Table 2) but varied in the four countries. Almost half of the Icelandic women in this cohort gave birth in water (48.1 %) compared to only 6.6 % in Sweden. No association between flexible sacrum positions and sutured injuries was found (OR 1.02; CI 95 % 0.86–1.21) or between flexible sacrum positions and SPT (OR 0.68; CI 95 % 0.26–1.79). Flexible sacrum positions were associated with fewer episiotomies after adjusting for potential confounders (primiparity, birth weight, transfer before birth and waterbirth) (OR 0.20; CI 95 % 0.10–0.54) (Table 4).

**Table 3:** Birth positions, waterbirth and flexible sacrum positions stratified by parity

	Total	Primiparas	Multiparas	Missing
Total	*N* = 2992	*N* = 524	*N* = 2422	*N* = 46 (1.5)
	n (%)	n (%)	n (%)	n (%)
Birth position				
Semi-recumbent	687 (23.0)	155 (29.6)	516 (21.3)	
Supine	238 (8.0)	49 (9.4)	185 (7.6)	
Lateral	420 (14.0)	64 (12.2)	351 (14.5)	
Birth seat/squatting	251 (8.4)	62 (11.8)	187 (7.7)	
All-fours	326 (10.9)	45 (8.6)	278 (11.5)	
Kneeling	737 (24.6)	100 (19.1)	629 (26.0)	
Standing	216 (7.2)	24 (4.6)	190 (7.8)	
Missing	117 (3.9)	25 (4.8)	86 (3.6)	
Flexible sacrum^a^				
Yes	1950 (65.2)	295 (56.3)	1635 (67.5)	
No	925 (30.9)	204 (38.9)	701 (28.9)	
Missing	117 (3.9)	25 (4.8)	86 (3.6)	
Waterbirth				
Yes	952 (31.8)	186 (35.5)	755 (31.2)	
No	2031 (67.9)	336 (64.1)	1660 (68.5)	
Missing	9 (0.3)	2 (0.4)	7 (0.3)	

^a^kneeling, all-fours, standing, squatting, birth seat, lateral

**Table 4:** Risk factors for different types of perineal trauma

	OASIS		Episiotomy		Sutured injury		Total perineal trauma	
	Crude OR	Adjusted OR	Crude OR	Adjusted OR	Crude OR	Adjusted OR	Crude OR	Adjusted OR
Primiparity^d^	8.19 (3.21–20.89)	9.90 (3.63–26.98)^c^	15.92 (6.79–37.30)	10.84 (4.28–27.45)^c^	2.74 (2.25–3.33)	3.07 (2.48–3.81)^c^	3.25 (2.66–3.97)	3.60 (2.89–4.49)^c^
Birthweight > 4000 g ^d^	1.93 (0.79–4.75)	2.87 (1.07–7.75)^a^	0.75 (0.31–1.85)	1.58 (0.59–4.18)	1.30 (1.10–1.54)	1.48 (1.24–1.77)^c^	1.32 (1.11–1.56)	1.53 (1.28–1.83)^c^
Transfer before birth	2.42 (0.71–8.30)	0.46 (0.06–3.59)	11.02 (5.31–22.84)	3.98 (1.72–9.22)^b^	1.33 (0.99–1.79)	0.91 (0.65–1.28)	1.60 (1.19–2.15)	1.01 (0.71–1.42)
Country	1.19 (0.70–2.03)	1.07 (0.60–1.90)	1.28 (0.82–2.01)	1.27 (0.78–2.09)	1.45 (1.32–1.58)	1.42 (1.29–1.56)^c^	1.47 (1.34–1.61)	1.44 (1.31–1.59)^c^
Flexible sacrum positions^e^	0.71 (0.29–1.75)	0.68 (0.26–1.79)	0.20 (0.10–0.44)	0.20 (0.10-0.54)^b^	0.89 (0.76–1.04)	1.02 (0.86–1.21)	0.84 (0.71–0.98)	0.96 (0.81–1.14)
Waterbirth	1.30 (0.54–3.16)	0.99 (0.36–2.73)	0.41 (0.16–1.06)	0.36 (0.13–1.03)	1.19 (1.02–1.39)	1.01 (0.85–1.20)	1.19 (1.02–1.39)	1.01 (0.85–1.21)

^a^ < 0.05

^b^ < 0.01

^c^ < 0.001

^d^adjusted for birthweight, transfer, flexile sacrum positions, waterbirth, country

^e^adjusted for parity, birthweight, transfer, waterbirth

## Discussion

The major finding of this study is a low prevalence of SPT and episiotomy which did not differ between the countries. The women in this cohort used a variety of birth positions and one third of them gave birth in water. No association was found between flexible sacrum positions and SPT or sutured injuries. Episiotomy was associated with giving birth in a non-flexible sacrum position.

The prevalence of SPT and episiotomy in this study is in line with previous research [21, 24, 38] and adds to the growing body of evidence regarding positive maternal outcomes and low levels of intervention for women with low risk choosing to give birth outside the hospital. Furthermore, the prevalence of SPT and episiotomy in this study did not differ between the countries, which is interesting considering the observed differences between the Nordic countries (2.3 to 4.2 %) [9–11]. Stating what prevalence of SPT should be considered to indicate good quality of care is problematic and has been discussed [39]. A low prevalence of SPT could be due to successful interventions during the second stage. On the other hand when midwives and obstetricians focus on assessment and classifying perineal injuries, the detection rate of SPT often increases [39, 40]. A prevalence between 1.0 and 3.9 % has been suggested to be a realistic target in high-risk units [39] but what rate is reasonable in a low-risk setting is not known. A lower prevalence of SPT could be expected in a low-risk setting such as home birth where fewer of the obstetrical interventions associated with SPT are present. Stedenfeldt et al. (2014) have shown that the greatest reduction in sphincter injuries after an educational program for midwives and obstetricians took place among low risk-births (i.e., second child, birth weight <4000 g and spontaneous birth with the baby in the occiput anterior position) [41].

The women in this study used a variety of birth positions and the majority used flexible sacrum positions. A recent review of the literature reports physical and psychological benefits for women when they give birth in an upright position of their choice [42] but the position assumed by women during birth is influenced by several complex factors. Upright birth positions occur more often within certain birth settings, such as birth centers and home [28, 29]. The midwife’s preference [43] as well as cultural values may influence the position for birth [34, 42]. It is not possible in this study to determine whether midwives influenced the position for birth but the variation in positions used suggests that women had the opportunity to choose position themselves. Although the number of SPT in this study was low, with only 21 detected cases, no generalizations can be drawn. However, none of the birth positions used – supine, upright or flexible sacrum positions – was associated with SPT which is in line with meta-analyses of the subject [34]. This indicates that midwives were skilled in attending women in different birth positions and it is also in line with the current evidence suggesting that women should be encouraged to give birth in the position most comfortable for them [34].

Flexible sacrum positions were associated with fewer episiotomies. To our knowledge and according to the midwifery literature [44, 45], midwives are taught to perform an episiotomy in the lithotomy or semi-recumbent position. This could imply that this finding is confounded by indication. If a midwife finds it necessary to perform an episiotomy, she will ask the woman to change position from a flexible sacrum position to a non-flexible sacrum position (semi-recumbent or supine). However, when looking at which birth position women had when the episiotomy was cut, 9 (30.0 %) of the 30 episiotomies were performed in a position other than the semi-recumbent or supine: 7 in the lateral position, 1 in squatting and 1 in the all-fours position. Five of the episiotomies were performed in water.

One limitation of this study is the lack of information regarding midwifery practices during the second stage to prevent perineal injuries, as well as midwives’ experience and training in assessing and suturing perineal injuries. There is evidence that perineal injuries are often misclassified [46] by both midwives and obstetricians. A second examiner and educational workshops have been shown to improve diagnosis and the appropriate classification of perineal trauma [46, 47]. Information regarding whether a rectal examination has been performed would be of value in further studies.

Another limitation is that midwives in four different countries entered the data. However, using the same protocol limits the classification bias. Midwives who assist women at home births are usually employed within the health care system and are used to recording this type of data, which is similar to the data entered in hospital records. The strength of this study is that the majority of women opting for a home birth and all the midwives assisting with home births on a regular basis in Norway, Sweden, Denmark and Iceland were identified and agreed to participate in this study. According to Blix et al. [48] the original cohort of 3068 women is suggested to cover 80–90 % of the planned home births in the four Nordic countries.

It is important to evaluate perineal outcome in relation to birth setting, since perineal injuries are associated with short- and long-term morbidity for women [49]. Home births in Norway, Sweden, Denmark and Iceland are not always registered and it is not possible to access the data specific to this study from the medical birth registers. The population studied consists of healthy women, giving birth without many of the interventions associated with modern obstetrics. Further studies are needed to assess long-term consequences of childbirth, such as urinary incontinence, dyspareunia, anal incontinence and the prevalence of prolapse in women giving birth at home. It would be of interest to study the impact of physiological birth on the pelvic floor in this group of women since they receive low levels of obstetric interventions.

## Conclusion

A low prevalence of SPT and episiotomy was found among women opting for a home birth in four Nordic countries. Women used a variety of birth positions and a majority gave birth in flexible sacrum positions. No associations were found between flexible sacrum position and SPT. Further studies are needed to assess the long-term consequences related to perineal injuries for women giving birth at home.
